# An extensive dust storm impact on air quality on 22 November 2018 in Sydney, Australia, using satellite remote sensing and ground data

**DOI:** 10.1007/s10661-022-10080-1

**Published:** 2022-05-14

**Authors:** Ali A. Attiya, Brian G. Jones

**Affiliations:** 1 Atmospheric Science Department, Science Faculty, Mustansiriyah University, Baghdad, Iraq; 2 School of Earth, Atmospheric and Life Sciences, University of Wollongong, Wollongong, NSW 2522 Australia

**Keywords:** Dust storm, Synoptic data, PM10, Dust sources, HYSPLT, Trajectory, MODIS image, Remote sensing

## Abstract

Recurrent dust storms represent a significant concern in Australia because of their related hazards and damages since particulate matter (PM) has harmful impacts on the environmental, health and economic sectors. The particulate matter may be released from natural sources and human activities. The major part of natural particulate matter is emitted into the air by wind erosion processes from desert and semi-desert areas at the world scale. A huge dust storm crossed over several areas of New South Wales (NSW), Australia, including the Sydney region on 21–22 November 2018 and decreased the horizontal visibility to less than 1 km for 22 h. This study examined the synoptic weather conditions, and assessed the air quality and identified the source and transport trajectory of the dust storm over Sydney using ground and satellite remote sensing data. PM10 (< 10 μm) concentrations were obtained from selected air quality monitoring sites operated by the Environmental Protection Agency in NSW. The highest hourly concentration of PM10 (578.7 μg/m^3^) was recorded at Singleton in the Hunter Valley, while concentrations in Sydney ranged from 480 to 385 μg/m^3^, well above the standard air quality level in Australia (50 μg/m^3^ per 24 h). The HYSPLIT back trajectories of air parcels suggest that the potential sources of the dust episode originated from the Lake Eyre Basin and northeast South Australia, the Mundi Mundi plains west of Broken Hill, Cobar and the grazing lands and the red sandplains in northwestern NSW. It then travelled towards the east coast. These long-range airflows transported suspended dust particles, raising air quality to hazardous levels (elevated PM10 levels) over most areas of NSW. The results from the HYSPLIT model for dust movement are confirmed by MODIS satellite images. Many areas of NSW experienced this intense dust storm due to northwest wind generated by the low-pressure systems and cold fronts over South Australia and many parts of western NSW as it moved eastward.

## Introduction

**Fig. 1:**
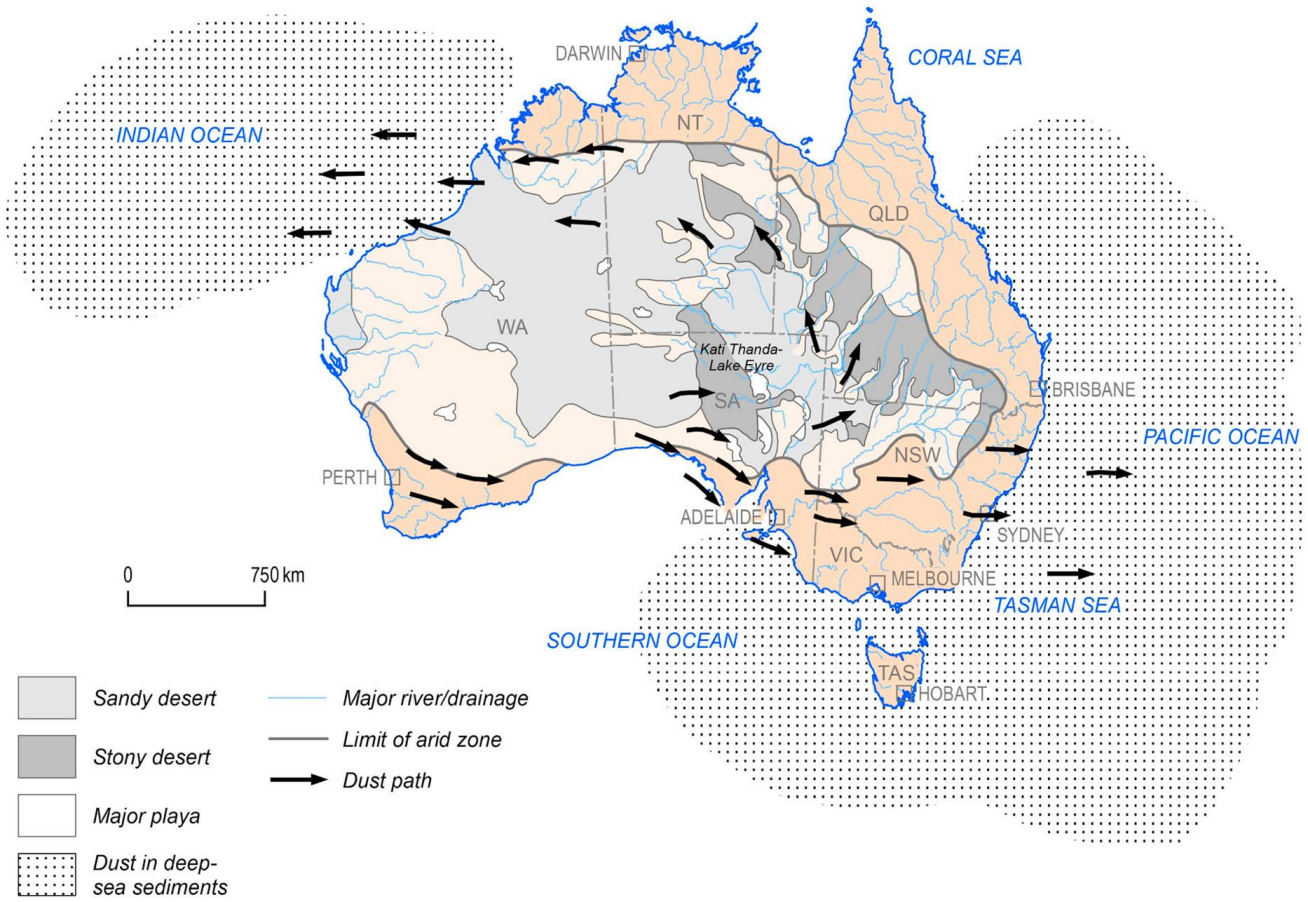
Australia map displaying the two principal areas of dust emissions in Australia, the main prevalent wind trends and the wide desert regions (from Blewett, 2012)

Dust plumes in the natural resource could be large source regions of suspended dust (Kuo & Shen, 2010). Most dust storms are emitted by arid and semi-arid areas (Shao, 2008) where annual precipitation is below 200 mm. Such dust belt areas (Prospero et al., 2002) include China, south and central Asia, the Middle East and the west coast of North Africa (Escudero et al., 2006). On a global scale, Australia represents the third largest source of dust emissions after North Africa (7%) and Asia (20%; Ginoux et al., 2004). Australian dust can be generated from any region on the continent and the biggest percentage of Australian dust is exported to two major areas — the Tasman Sea in the east and the Indian Ocean opposite the northwest coast of Western Australia (Fig. 1; Bowler, 1986; Sturman & Tapper, 1996).

Dust events are a natural phenomenon that occur regularly in Australia and are widespread in the internal desert areas (e.g. Sturt, Great Victoria, Gibson and Simpson Deserts; Knight et al., 1995; McTainsh et al., 2005). The alluvial channels and dry lake beds in the Lake Eyre Basin are significant sources of dust (Bullard & McTainsh, 2003; Shao et al., 2007) and represent the eighth major dust source area at a global scale (McTainsh et al., 1998; Middleton, 1984; Washington et al., 2003). This basin represents about 1/6 of the total area of Australia continent (Strong et al., 2011) and most dust events occur between September and December (late spring to early summer; Ekström et al., 2004). Australian dust events also originate from agricultural regions that have been exposed to long periods of drought in New South Wales, Victoria, Queensland and western South Australia, as well as from the Broken Hill and Cobar mining areas. Large dust storms may impact wide regions and lead to economic losses at local and national levels (Wang et al., 2006; Stefanski & Sivakumar, 2009; Tozer & Leys, 2013) since they can impact on climate, human health, socio-economic and environmental issues (Gerivani et al., 2011). Atmospheric dust activity represents an important issue and a major concern because of its influences on the economy and public health sectors across eastern Australia, including Brisbane, Canberra and Sydney, during the last few years.

Wind can transport dust and various other pollutants for long distances and can affect large down-wind areas (Shinn et al., 2000; Wang et al., 2007). In central Australia (NSW and Queensland) dust events are mainly generated in September–January (spring and early summer seasons), while in southern Australia (Victoria and southern NSW) dust storms mainly occur in December to March (summer season; McTainsh et al., 1998). Sturman and Tapper (1996), using data from 95 climatic stations, studied the relationship between dust storms and synoptic conditions in Australia. They found two main categories of dust events: (a) spring–summer dust events that are affected by limited precipitation and the occurrence of non-rainfall air cold masses over central and southern Australia; and (b) summer-autumn dust events that are related to long periods of drought during the year over the coasts of western and southern Australia.

The capital cities of Australia have been exposed to several dust storms during the past four decades. Brisbane and Sydney were affected on 23 October 2002 while the 23 September 2009 dust storm (red dawn event) affected the east coast cities in Australia — both were documented as severe events (Leys et al., 2011; McTainsh et al., 2005). Other prominent dust storms happened on 1/02/1983 and 24/05/1994 in Adelaide (Raupach et al., 1994; Williams & Young, 1999), on 25/05/1994 in Sydney (Raupach et al., 1994), on 1/12/1987 in Brisbane (Knight et al., 1995) and on 8/02/1983 in Melbourne (Raupach et al., 1994). During these dust storms, Brisbane and Sydney have been covered by the dust clouds for about 24 h before they move east over the Tasman Sea region (Aryal et al., 2012). Indeed, the 23 September 2009 dust storm transported about 300,000 tonnes out to sea between Newcastle and Wollongong (Albian Park) at a maximum rate of 75,000 t/h (Tozer, 2012) out of a total soil loss of > 2.5 Mt (Leys et al., 2011). Because most dust plumes moved across the Lake Eyre basin and rural territories, dust particles were thought to consist of mainly of desert sediment and surface soils from farmland. Some researchers examined the compositions of dust and tried to identify likely dust source regions in Australia through mineral analysis of dust particles in the air, surface material properties and transport modelling of dust (Aryal et al., 2012; Knight et al., 1995; Li et al., 2010; Radhi et al., 2010a, b).

The present study aims to examine synoptic weather conditions and the variability of PM10 concentrations before, during and after a major dust storm in November 2018 to assess changes in air quality. These raised PM10 values are compared with normal levels for selected monitoring stations sites in the Sydney region. The identification of the November 2018 dust storm source and its transport trajectory is assessed using ground and satellite remote sensing data.

## Dust entrainment and transport

The long-range dust transport (dust entrainment process), dust generation and the temporal variability of dust emissions in paleo-environments were documented in a geomorphic literature review by Marx et al. (2018). A dust storm that originated in the Gobi and Taklamakan Deserts over China and moved towards Japan and Korea during March 2010 was examined by Chen et al. (2017) using the available ground and satellite data. Chen et al. (2018) examined dust transport from the Taklamakan (Asia) and Sahara-Sahel (North Africa) Deserts towards the east and west seaboards of the USA during two dust storms in 2015 using the ground and satellite data. Wain et al. (2006) investigated a dust storm that occurred in central and eastern parts of Australia in October 2002 and found that a transport model can be a good tool to predict the location of the dust cloud. Other Australian studies have also applied transport models to determine source regions and transport pathways of dust (De Deckker et al., 2008, 2014; McGowan & Clark, 2008 O’Loingsigh et al., 2017). The HYSPLIT (Hybrid Single-Particle Lagrangian Integrated Trajectory) model can be used to investigate such dust events in any part of the world by analysis of the backward pathway of air parcels at various elevations over time (Draxler & Hess, 1998). For example, the HYSPLIT model trajectories and satellite data were used to examine the 2010 dust storm at between ~ 6- and ~ 8.5-km elevations over South America (Draxler et al., 2010).

Moderate Resolution Imaging Spectroradiometer (MODIS) satellite images from Aqua and Terra sensor observations were used by Bullard et al. (2008) to monitor dust storms during 2003–2006 above the Kati Thanda (Lake Eyre) area in Australia. They recognised 529 dust events and identified various dust source areas (29% from playa lakes, 30% from flood plains and alluvial deposits and 37% from dune fields). They showed river floodplains that are exposed to flooding by muddy water are likely dust sources during subsequent dry periods (Bullard et al., 2008; McTainsh & Pitblado, 1987). Bullard et al. (2008) also indicated that dry lake margins account for up to 14% of dust plume emissions.

Capizzi et al. (2015) showed that particulate matter (PM) is the most dispersed pollutant in major cities. PM concentrations can impact public health and environmental systems and they represent a big concern in urban regions because of their hazards (Aryal et al., 2012). Dust storms travelling over interior cities and the east coast of Australia can influence air quality in these population centres by decreasing horizontal visibility and causing diseases (Chan et al., 2005; Leys et al., 2011; McKendry et al., 2001). Particulate matter can be used as indicator of air pollution when it is < 10 μm in diameter (PM10) in a specific region (Chan et al., 2005; Künzli et al., 2000; Lim et al., 2010; Lu, 2002; Ragosta et al., 2006). Other studies indicate that PM10 concentrations represent a major concern in several areas around the globe where they are exposed to recurrent dust storms, e.g. Spain, Australia, Korea, Japan and China (Ekström et al., 2004; Kim et al., 2001; Lyamani et al., 2005; Vanderstraeten et al., 2008; Watanabe et al., 2011; Xie et al., 2005). The suspended particulates which have smaller diameters (e.g. PM2.5, PM1) also have adverse impacts on air quality (Lanzafame et al., 2015) and public health (Caramagna et al., 2015). PM concentrations may originate from soils by wind erosion (e.g. agricultural and rural lands, as well as urban soils) and be transferred for long distances. However, PM is also produced from industrial, commercial and vehicular contributions (mechanical disturbance) in civilian regions (Aryal et al., 2008; Lee & Lee, 2008). The grain size of dust reduces as the distance downwind increases because larger particulates like feldspar and quartz are deposited faster than finer clay minerals (Van Der Does et al., 2016). However, mineral dust particles > 75 µm have been discovered much farther away from their source regions than predicted because of uplift and turbulence processes in particular thermal convective systems (Van der Does et al., 2018). Dust particles can directly affect the solar radiation balance by absorbing and scattering solar radiation or indirectly affect the hydrological cycle of the atmosphere by acting as condensation nuclei and changing the cloud forming characteristics, and thus they can affect the climate system (IPCC, 2007; Rotstayn et al., 2009). The scattering and absorption processes of solar radiation caused by dust storms may also affect air temperature (Miller & Tegen, 1998).

PM, by increasing air pollutants levels, has caused health problems such as respiratory, cardiovascular and chronic lung illness and increased hospital admissions and the mortality rate during the last decade (Glinianaia et al., 2004; Maisonet et al., 2004). Some research has revealed a correlation between PM concentration and health problems (e.g. lung, breathing, heart attack and stroke respiratory diseases) as well as increasing incidents (3 to 6%) and mortality rates (1%) when the PM concentration is > 10 μg/m^3^ (Donaldson & MacNee, 2001; Goldberg et al., 2006; Kan & Chen, 2003; Mehta et al., 2013; Ostro et al., 1999). Jayaratne et al. (2011) examined the particle size distributions and PM10 concentrations observed in Brisbane city during a dust storm on 23 September 2009 and compared them with normal days. The dust storm in this city, situated about 1000 km north of Sydney, reached a maximum at mid-day when the hourly mean PM10 and PM2.5 concentrations were about 6460 and 814 μg/m^3^ with the PM10 concentration forming around 68% of the total mass.

Daily PM10 values were reported of around 2700 at Zhurihe in northeast China and 7414 µg/m^3^ at Tazhong in west China between 2001 and 2006, an average of 364 µg/m^3^ over 24 h in Lubbock, TX, in the USA, and 2700 µg/m^3^ in Kuwait (Draxler et al., 2001; Gong & Zhang, 2008; Lee et al., 2009; Wang et al., 2008). Hourly air quality data from the Environment Protection Authority in New South Wales and Queensland for four coastal cities during the October 2002 dust storm event were used to notify public health and amenity organisations in the study cities (Chan et al., 2005).

Some research studied the relationship between atmospheric conditions and dust events in Australia (Ekström et al., 2004; Raupach et al., 1994; Strong et al., 2011). The relationship between cold fronts and dust events in the Tarim Basin in northwest China was also examined by Aoki et al. (2005). An understanding of the relationship between atmospheric conditions and dust events provides a better understanding of dust entrainment mechanisms by displaying how the air parcels behind cold fronts are the main cause of dust events at the synoptic scale. Dust transport pathways in Australia are related to the movement of air cold masses and associated changes in air temperature and wind direction that split the eastward movement of two anticyclones as shown by the wind model of Sprigg (1982).

The relationship between dust storms and associated meteorological patterns that could cause dust entrainment in the lower Lake Eyre Basin between 2005 and 2006 was examined by Bullard et al. (2008), Strong et al. (2011) and Baddock et al. (2015). They indicated that Australian dust entrainment has a distinct seasonal distribution from late spring to mid-summer in the northern part of Australia and the full summer season in southern Australia. This is caused by the heating process during summer in the southern hemisphere leading to a shift in pressure systems and troughs as air cold masses progress through spring and summer.

Dust events and meteorological observations in the whole of Australia between 1957 and 1984 were compared by McTainsh and Pitblado (1987). The results revealed that more than 5 dust storms per year were recorded in many regions of northern Western Australia, south-central Queensland and central Australia, as well as in the Mallee area and Murrumbidgee River. Climatic factors, vegetation and soil erosion were the three main factors influencing dust events. However, climatic conditions were more significant on a large scale than the effects of vegetation and soil erosion. McTainsh et al. (1989) suggested that there were non-linear correlations among rising dust events and drought periods.

## Study site

**Fig. 2:**
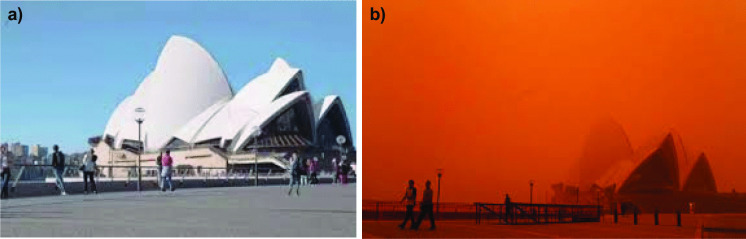
Showing pictures of **a** before and **b** during the huge dust storm on 22 November 2018 in Sydney, which turned skies orange and covered the Opera House in a dark red haze

Sydney, the capital of NSW, is located on the east coast of Australia and is the largest (area about 12,370 km^2^) and most populous city (about 5.23 million in 2018) in Australia. Sydney (33°51′54 S, 151°12′34 E) surrounds Port Jackson and extends around 70 km to the Royal National Park in the south, Macarthur in the southwest, the Blue Mountains in the west and Hawkesbury River to the north. Southern and northern Sydney have different concentrations of air pollutants because the sea breeze generally comes from the northeast and pollutants can be trapped in the low-lying areas in western Sydney. Therefore, the Sydney area was divided into southwestern, northwestern and eastern sub-areas according to the Office of Environment and Heritage. The two pictures before and during the huge dust storm on 22 November 2018 in Sydney are shown in Fig. 2.

## Datasets and methodology

**Fig. 3:**
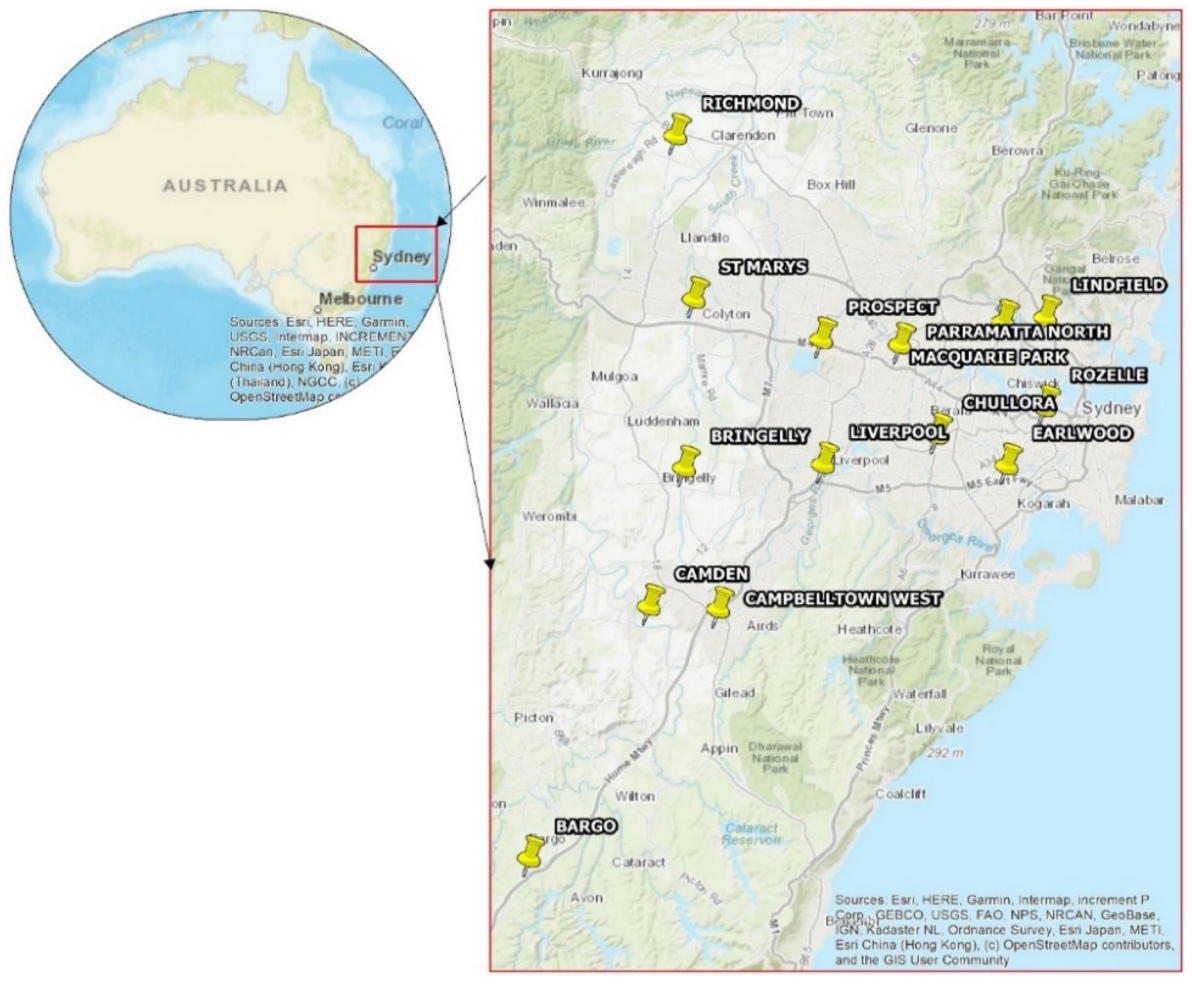
Monitoring stations recording PM10 concentrations in the Sydney area (yellow pins)

The air quality standard exceedances before, during and after the 22 November 2018 dust storm in the Sydney region were monitored by fourteen air quality monitoring stations (Fig. 3) operated by the Office of Environment and Heritage (OEH) in NSW. The hourly mean particulate matter (PM10) data was obtained by the OEH in order to assess air quality within the Sydney metropolitan region. The synoptic weather charts and climatic data (relative humidity (RH%), air temperature, wind speed and direction) were obtained from the Bureau of Meteorology (BOM) in Australia.

The Hybrid Single-Particle Lagrangian Integrated Trajectory (HYSPLIT) model was applied to determine the sources and pathways of the dust plume using meteorological data (resolution 1° × 1°) from the http://ready.arl.noaa.gov/HYSPLIT.php website. The low resolution of the meteorological field causes some uncertainty in the model. The HYSPLIT model contains three kinds of pathways that could be calculated — ensemble, matrix and normal. The ensemble pathway is used to determine multi-trajectories from one location through all potential aberrations in three coordinates (X, Y and Z) since it decreases the uncertainty caused by the meteorological resolution (Draxler & Hess, 1998). The HYSPLIT backward trajectory model calculated air parcel movement over a duration of 72 h at 100-m, 500-m and 1000-m elevations above ground level (AGL) to identify possible sources and transport trajectories from dust hotspots.

In order to support the findings of the HYSPLIT model, MODIS (Moderate Resolution Imaging Spectroradiometer) imagery has been employed in the current research. MODIS satellite images provided by NASA represent a useful tool to monitor and determine dust plume movement utilizing optical images based on the scattering and radiation properties of particulate matter.

## Results and discussion

The Bureau of Meteorology reported 2018 as the warmest year on record for maximum and mean temperatures in New South Wales with the entire state being drought-declared in August. Extreme heat featured throughout 2018, especially in January, April and December. Rainfall was 40% below average, being the driest year since 2002 and the sixth driest on record (BOM 2019). Spring brought warm dry windy conditions associated with the passage of cold fronts in September and November. Rainfall totals continued to be below average overall, although some areas were wet, with heavy falls in November including around Sydney (BOM 2018e). Extensive dust storms continued throughout the state as most of the State remained drought-affected until the end of 2018.

The worst of the September 2018 dust storm hit Sydney on the morning of 22 November. The conditions were similar to those that led to the major dust storm in September 2009, which also blanketed Sydney as well as much of NSW (Bullard et al., 2008; Leys et al., 2011). According to the Bureau of Meteorology, the dust storm on 22 November 2018 extended from the Victorian border, through Canberra and up towards Queensland (more than 500-km wide).

### Synoptic conditions during the November 2018 dust event

**Fig. 4:**
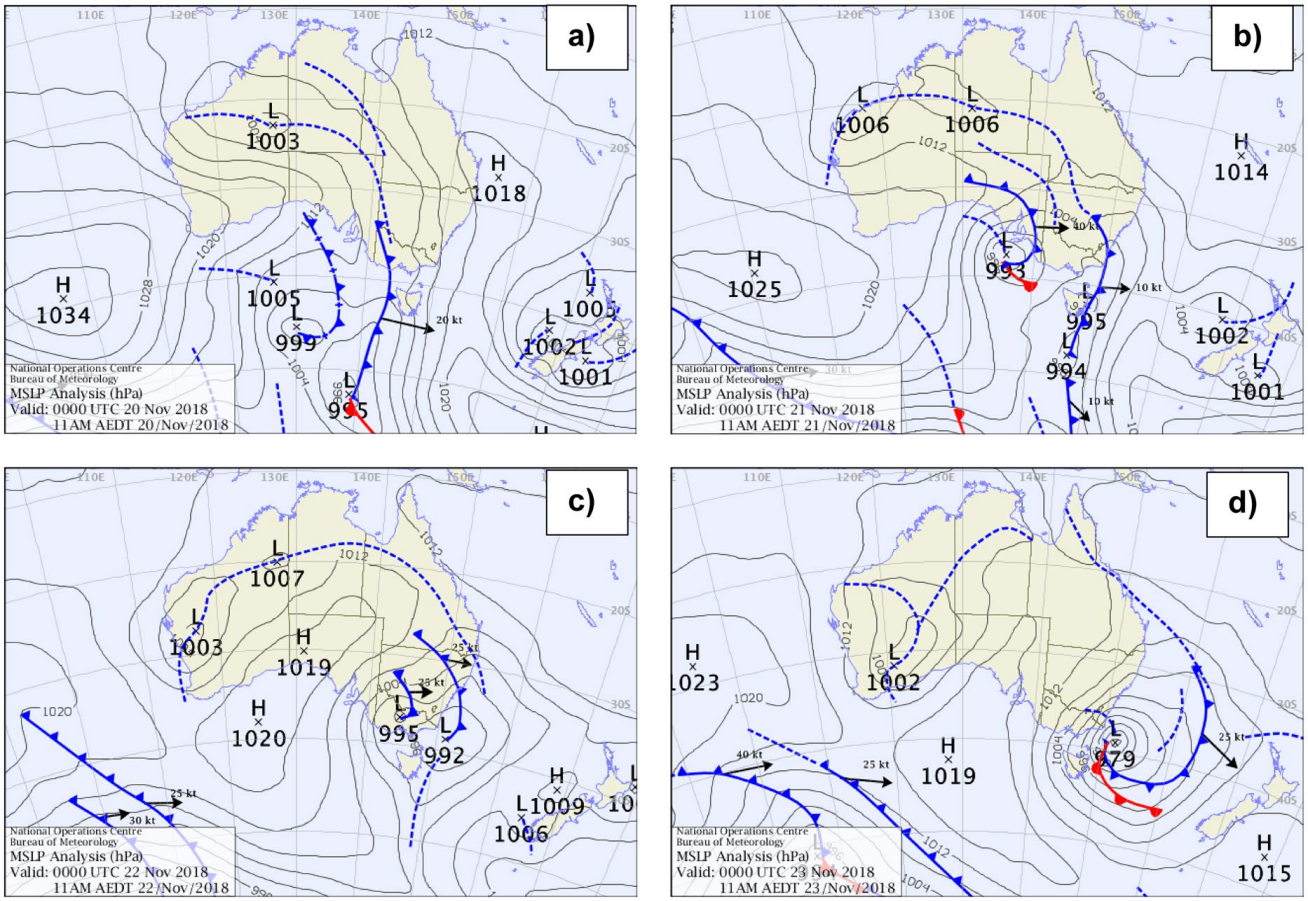
Synoptic charts displaying the strong western wind produced by low-pressure systems and two cold fronts on 21 November seen before, during and after the large dust storm on 22 November 2018. **a** 20 November 2018 00:00 UTC; **b** 21 November 2018 00:00 UTC; **c** 22 November 2018 00:00 UTC; **d** 23 November 2018 00:00 UTC. http://www.bom.gov.au/australia/charts/archive/index.shtml

On 20 November 2018, two separate large low-air pressure cells caused a trough of cold low-pressure air to extend over a wide area of Australia (Fig. 4a). As the low-pressure cells moved across NSW, it generated prefrontal western wind over western New South Wales on 21 November and over the east coast of Australia on 22 November 2018. On 22 November, the low-pressure systems met on the east coast of NSW; the air cold mass length increased while the low-pressure trough length decreased (Fig. 4b–d).

As the air cold mass and low-pressure trough moved from west to east (Fig. 4a–d), surface wind paths changed considerably. Using the wind data from the Australian meteorological stations, strong surface winds initially came from the south-west but this changed as the cold air mass moved across and the area and the wind then came from the west. This pattern of surface winds related to low-air pressure troughs and air cold masses is consistent with wind patterns over the longer-term, as shown by many researchers during the previous seven decades (e.g. Sprigg, 1982; Strong et al., 2011). Similar synoptic patterns also occurred during 5 previous severe dust plumes through the Australian Dust Bowl between late 1930 and early 1940 (Loewe, 1943). The extreme surface winds above the wide area of South Australia and the much of western NSW lifted and transferred the dust plume towards the east coast of Australia.

### November 2018 dust event

The cold air front which moved through South Australia on 21 November 2018, along with strong winds originating from a low pressure system in the dry centre of Australia, moved dust from the Simpson Desert, western NSW, and the Strzelecki Desert and Lake Eyre Basin in South Australia towards Sydney and eastern NSW (Fig. 1). Weather conditions included an unstable atmospheric layer that caused dust particles to rise from surface soils into the atmosphere. The large movement of dust was due to the extreme drought conditions producing very dry soil that contributed to the amount of dust gathered by intense winds. The drought caused an increase in the frequency of widespread dust storms throughout New South Wales in 2018 compared with 2017.

**Fig. 5:**
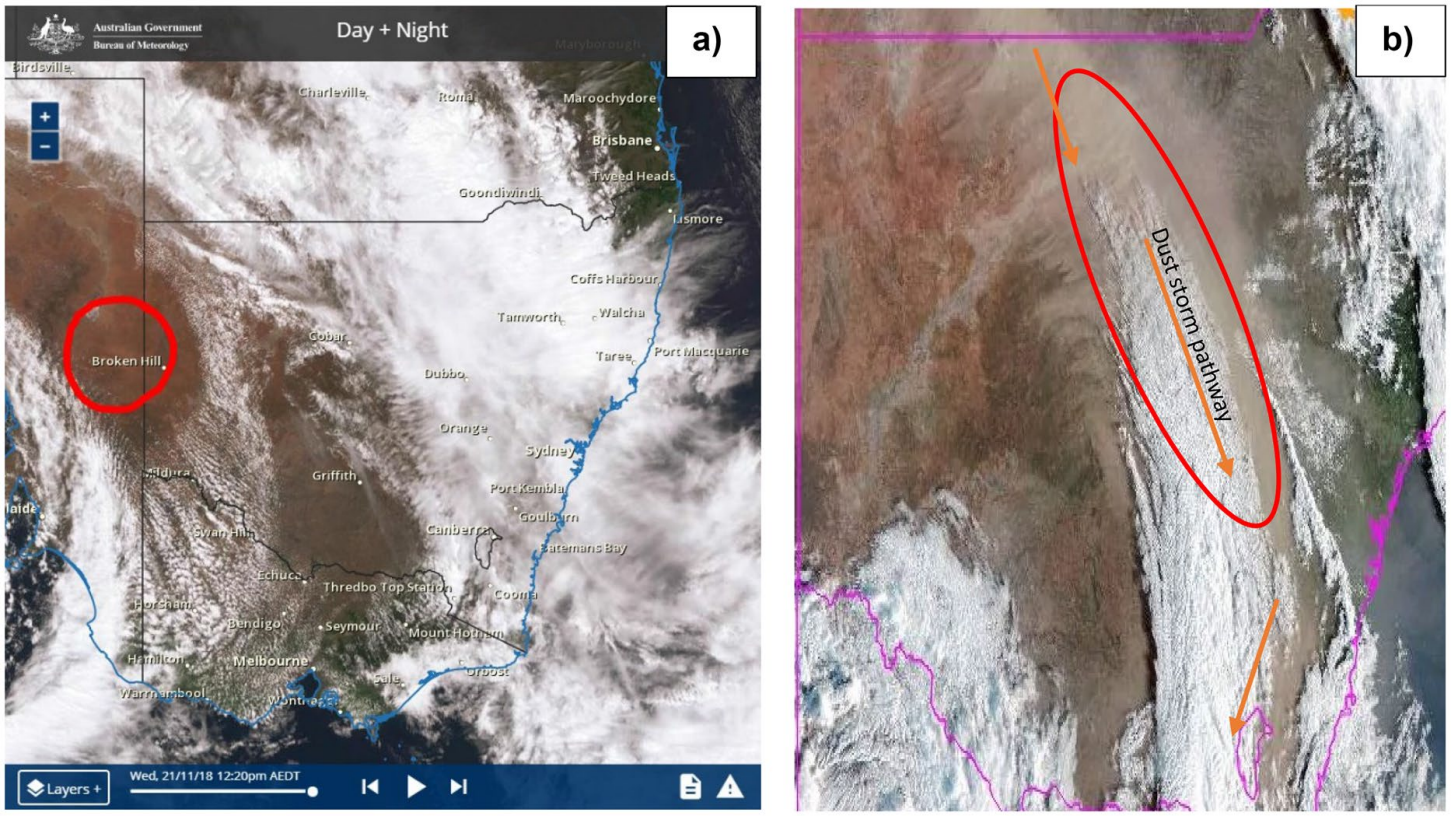
**a** Satellite image showing the dust emanated from eastern South Australia on Wednesday 21/11/2018 and travelled across New South Wales overnight; and **b** a satellite image showing the dust haze that engulfed Sydney and the eastern coast on Thursday 22 November 2018. Refer to Fig. 4 for the relevant synoptic weather charts for the Australia during 20–23 November at 12:00 UTC

During November 21 and 22 in 2018, the dust travelled over northern NSW, heading for the east coast. Observed surface wind velocities ranging from 6 to 12 m/s at 1 to 2 km above the ground. A satellite image from the Bureau of Meteorology for 12.20 pm on 21 November revealed post-frontal dust entrainment over South Australia, with the majority of the dust coming from dry lake beds. The low-pressure trough and cold front, which had been situated in South Australia the previous day, travelled towards the western parts of NSW including Broken Hill, the Riverina, the Central West, White Cliffs, Menindee, Brookdale, Griffith and Wagga Wagga. Drought conditions had dried the agricultural soil, which made it easier for the wind to pick up dust. Thus, the strong winds associated with the widespread cold front swept across drought-stricken parts of New South Wales and increased the size of the dust storm. The dust storm, which stretched about 500 km from north to south, reduced visibility to just metres in far western NSW, including at Darling River and Broken Hill. The dust travelled across NSW towards the east coast (Fig. 5), where the average surface wind velocity ranged from to 12.4 to 9.3 m/s with gusts up to 15 to 20.6 m/s observed in places such as Beresfield, Singleton and Muswellbrook. The dust storm arrived in Sydney on the morning of Thursday 22 November (Fig. 4c) leading to decreased visibility (below 500 m) causing traffic disturbances and at least 40 flights were cancelled or delayed at Sydney Airport. The gusty winds which brought the dust forced the airport to operate on a single runway delaying both international and domestic flights. By lunchtime, the impact of the dust was obvious across iconic locations like the Opera House (Fig. 2b), Sydney Harbour Bridge and Bondi Beach, while conditions in western Sydney had also deteriorated.

**Fig. 6:**
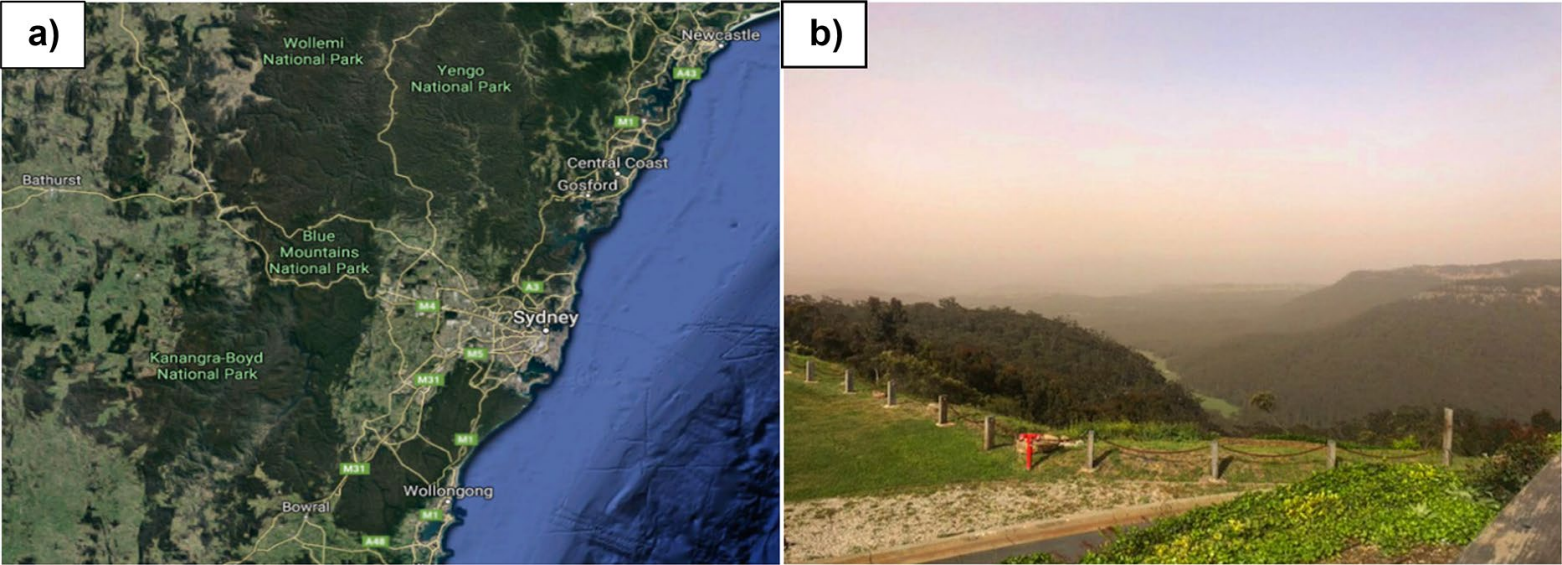
**a** Forest and cleared areas around Sydney and the Blue Mountains in NSW, and **b** the dust storm starting to move over the Blue Mountains at 1.35 pm on 21 November 2018

PM10 distributions during the 22 November dust storm in Sydney started with normal concentrations in the atmosphere (Fig. 5a) before suspended dust particles moved in from the west and progressively travelled eastward across Sydney towards the east coast (Fig. 5b, c, d, e). This movement was caused by the strong westerly winds (up to 90 km/h) related to the passage of low-pressure systems and air cold fronts over New South Wales and off the east coast (Fig. 8). The dust storm was initiated by a cold front that moved through South Australia on 20 November 2018, along with a low-pressure trough over New South Wales. Griffith in southwestern NSW experienced the dust storm on Tuesday 20 November 2018. This same widespread cold air mass transported the red dust across western NSW (an extensive dust storm with visibility < 1 km blanketed Broken Hill on 21 November at about 12.20 pm) towards Sydney some 1100 km to the east. This cold air mass moved past Orange and Bathurst towards Lithgow and at about 9.30 a.m. on the 22 November it arrived in the Blue Mountains (Fig. 6b) as it approached Sydney. The peak of the dust hit Sydney on midmorning 22 November 2018 reducing visibility to below 500 m (e.g. Fig. 2b). This affected flights at Sydney Airport (International and Domestic terminals) for most of the day according to Air services Australia. Later this cold air mass moved off the east coast of Australia with some of the dust being transported to New Zealand.

### PM10 concentrations

On 21–22 November 2018, most regions of New South Wales experienced very poor to hazardous air quality. Air quality was affected by long-range transport of dust particles from South Australia and the drought-affected regions of New South Wales during the passage of the cold front. PM10 24-h average levels exceeded the benchmark of 50 μg/m^3^ at 44 of the 47 ambient air quality monitoring stations in New South Wales. The Upper Hunter Valley at Camberwell 80 km northwest of Newcastle (Fig. 6a) recorded a maximum daily PM10 level of 243.9 μg/m^3^. However, the maximum hourly concentrations in the Upper Hunter occurred at 11 am in Singleton (578.7 μg/m^3^ with 9.8 m/s wind and 31.3% humidity) and Muswellbrook (532.9 μg/m^3^ with 8.6 m/s wind and humidity 35.7%). These regional centres are situated 140–180 km north of Sydney. The elevation (600–1189 m) of the Blue Mountains (Great Dividing Range, Fig. 6a, b) to the west of Sydney had a significant impact in decreasing the concentration of dust which arrived in Sydney (as previously suggested by Li et al., 2010, and Cohen et al., 2011). A similar decrease in the PM10 values in Wollongong (305.6 μg/m^3^), some 60 km south of Sydney, is due to the Southern Highlands to the west. Thus, the dust storm mainly moved into Sydney from the west.

**Fig. 7:**
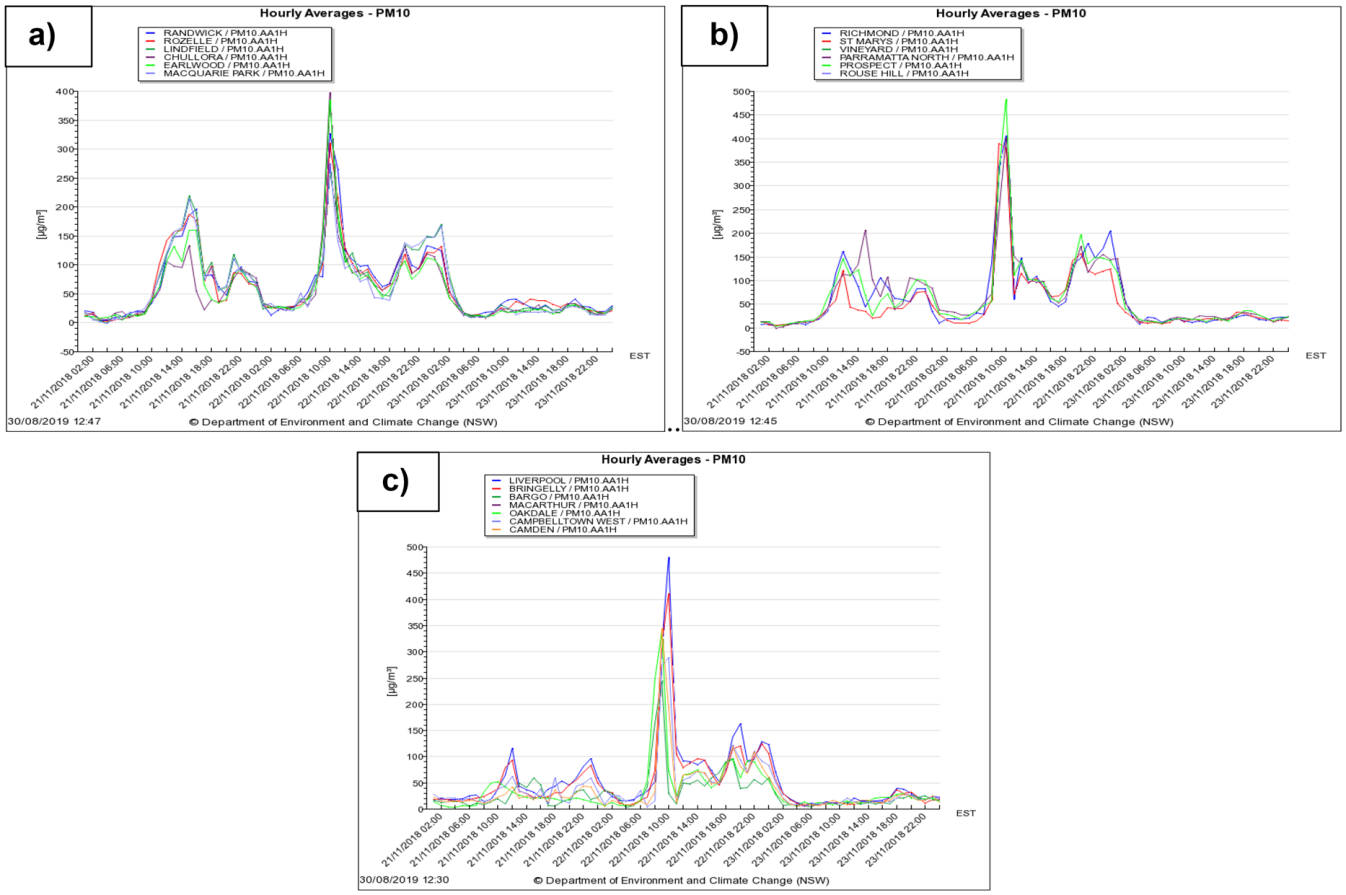
PM10 data before, during and after the 22 November 2018 dust storm in the **a** central-east, **b** northwest and **c** southwest Sydney regions

**Fig. 8:**
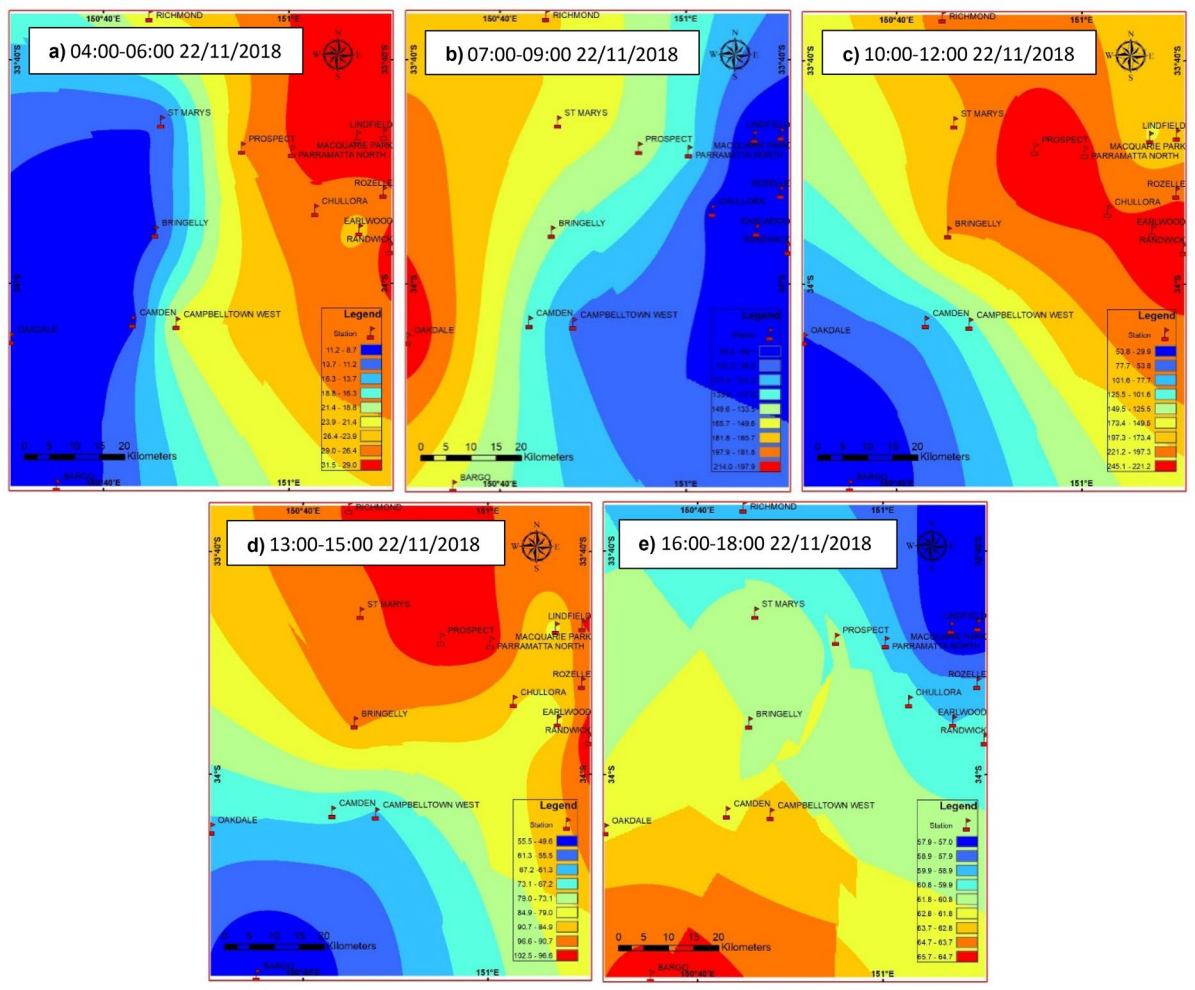
Distribution of PM10 values in µg/m^3^ before (**a**), during (**b**, **c**, **d**) and after (**e**) the 22 November 2018 dust event in the Sydney region. Note the different scales on all the maps

Early on 21 November 2018 morning, Sydney city had been exposed to humid and warm weather. The surface wind direction was northerly, the wind speed was around 2.5 m/s and humidity was about 60% with a temperature of 24.9 °C. The particulate matter (PM10) value was less than 50 μg/m^3^, which represents a typical air quality during 24 h in Australia. The PM10 value in Sydney increased gradually from 8 am on 21–11-2018 until by 9–10 am on 22–11-2018 it reached a range between 200 and 483 μg/m^3^ as wind velocities from the northwest and west-northwest increased to 8.3–10.3 m/s over different sites across Sydney. Figure 7 displays PM10 concentrations before, during and after the huge dust storm at different sites in Sydney, Australia. The maximum PM10 concentration (483.3 μg/m^3^) was in the Prospect area in northwestern Sydney at about 10 am on 22/11/2019 but high values were recorded throughout most of Sydney (see Table 1). PM10 levels decreased again to the standard level (< 50 μg/m^3^) on the next day. The large differences in PM10 values during the dust storm in Sydney displayed that surface wind directions had a significant impact on PM10 concentrations (Fig. 8).

**Table 1:** Wind velocity, humidity and PM10 concentrations in the Sydney area at 10 am on 22 November 2018

Location	Area	Wind velocity (m/s)	Humidity (%)	PM10 (μg/m^3^)
Prospect	NW	6.5	37.5	483.3
Richmond	NW	5	44.4	405.3
Parramatta North	NW	3.2	39.3	400.7
St Marys	NW	5.6	37.7	380.2
Liverpool	SW	6.7	35.1	480.0
Bringelly	SW	5.3	37.0	411.0
Campbelltown	SW	7.4	40.8	288.4
Chullora	Central	4.0	40.8	397.7
Eastwood	Central	3.8	36.7	385.0
Randwick	E	2.9	42.7	327.0

The local surface wind circulation at Sydney influenced dust particle dispersion over a 500-km area (Fig. 8b, c, d). A cloud of dust more than 1200-km long could be seen from the Victorian border, through Canberra and up to Queensland on 22 November 2018. The PM10 concentrations came back to acceptable levels in the atmosphere after the dust storm passed across Sydney over 24 h. The huge dust storm on 22/11/ 2018 with concentrations of PM10 up to 483.3 μg/m^3^ at Prospect in the northwest Sydney region had a much greater impact in Sydney than the 23/10/2002 dust event that had PM10 concentrations of 266 μg/m^3^ (Chan et al., 2005).

### Effects of the dust storm

The dust storm affected several coastal areas including Sydney, the Illawarra, Hunter Valley and Central Coast. The air quality between Sydney and the Hunter reached hazardous levels in North Parramatta, the Central Coast and upper Hunter region and air quality was close to hazardous levels in Sydney’s eastern suburbs. This storm led to breathing difficulties and conditions like asthma can be deadly since dust particles can get deep into people’s lungs and cause heart and lung conditions, asthma and emphysema to worsen. NSW Ambulance Assistant Commissioner Toney Gately said paramedics had responded to 90 cases of asthma or breathing difficulties from midnight to 6:00 am and then eight calls per hour with respiratory complaints throughout the day. Poor air quality continued into the morning of 23 November. The conditions had the same hallmarks of a major dust storm in Sydney on September 23, 2009, when there were also admissions to hospitals and respiratory problems (Ramachandran, 2009).

### Modelled sources and transport pathways of the dust event

**Fig. 9:**
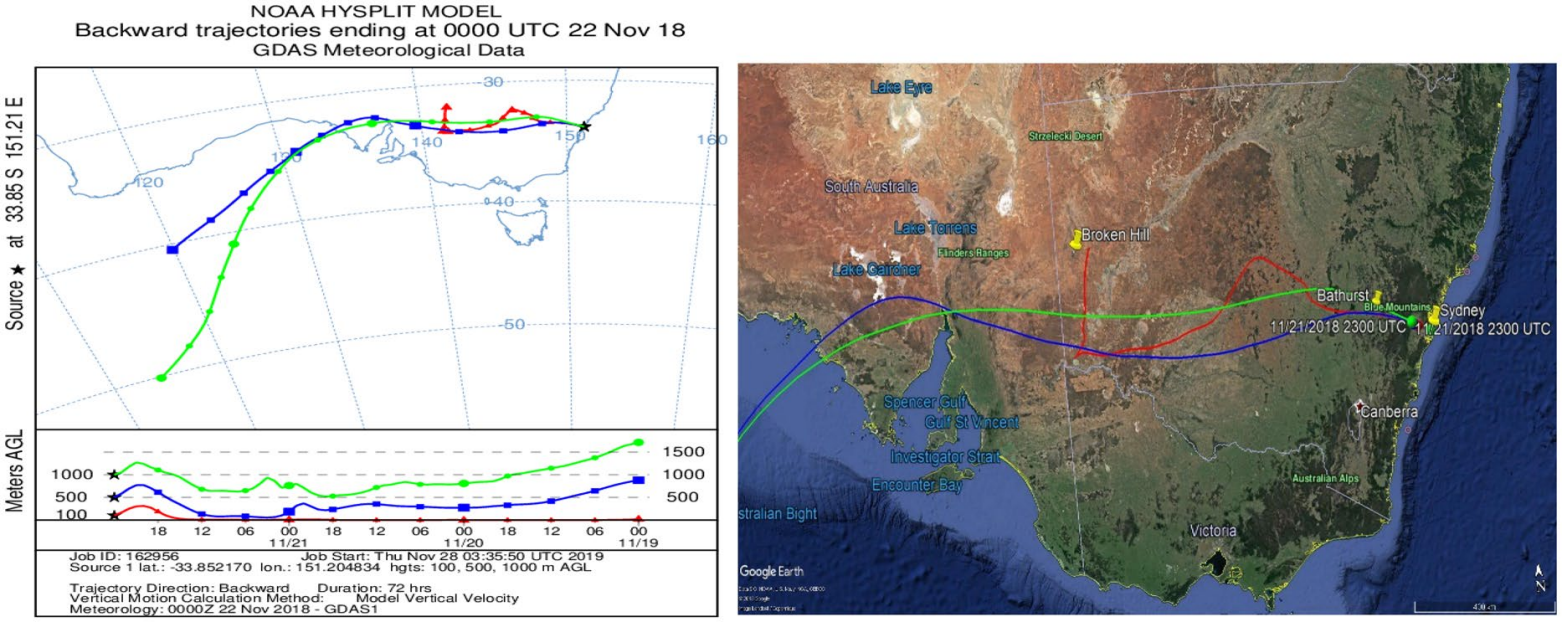
The HYSPIT back trajectories for the dust storm of 22/11/2018 superimposed on a Google Earth image

In the present study, potential sources and transport pathways of the dust storm were simulated using the backward trajectory of air masses from the HYSPLIT (Hybrid Single Particle Lagrangian Integrated Trajectory) model. The HYSPLIT model is a computer program utilised to calculate air-mass pathways and the dispersion or deposition of air pollutants. This approach was developed by the NOAA agency in USA and the Bureau of Meteorology in Australia. For the huge dust storm on 22/11/2018, based on the arrival time of the dust storm in Sydney, a backward trajectory simulation of the air parcel was began from 12 UTC on November 22nd 2018. The backward trajectories plots from the HYSPLIT model revealed that source region of dust storm was in South Australia (Lake Eyre Desert) and near the western border of NSW (Broken Hill) as shown in Fig. 9a. The height of the dust storm in Sydney was less than 500 m above the ground surface (AGL). In order to support the results of the backward trajectory of the air parcel by HYSPLIT, MODIS satellite data has been utilised to visually monitor the dust plume (Fig. 7). The optical imagery from the MODIS satellite records the scattering and radiation properties of dust particles and it was obtained from the NASA agency quick reply imagery. According to Figs. 7 and 9, on November 21, dust moved over Broken Hill and then gradually crossed above NSW to the northwest, southwest and central-east parts of Sydney before crossing the east coast of Australia. Thus, the MODIS satellite images confirmed the backward trajectory results of air parcels from the HYSPLIT model.

The results of HYSPLIT backward trajectories for the dust event are displayed in Fig. 9 to examine the dust storm trajectory and its sources on 22/11/2018, over duration 72 h and at elevations 100, 500 and 1000 m above ground level (AGL). In the HYSPLIT model, the source of the dust in the study area has been the dust hotspots based on Lake Eyre Basin in South Australia, the Broken Hill mining regions and grazing lands from western NSW towards Bathurst. The back trajectory of the air parcel situated at 100-m elevation in Sydney on 22 November had been at an altitude of below 100 m in the western region 72 h earlier where dust was entrained from around Broken Hill and the grazing lands to the south and east. The back trajectory of air parcel situated at 500-m elevation in Sydney had been at an altitude of around 100 m in the western region 24 h earlier. The higher air parcels between 500 and 1000 m come from South Australia (lower part of Lake Eyre Basin). The arrival time of a dust storm is related to the direction from which the air parcels arrive in the Sydney region. The lower air parcel (red trajectory) came from the northwest towards the east coast of Australia (Sydney) and moved at low altitude around the Blue Mountains. The other two air parcels moved at high elevations and passed over the Blue Mountains where their velocity was reduced by friction leading to deposition of the larger and heavier dust particles.

Figure 9 shows that the HYSPLIT backward trajectories of air masses travelled from the Lake Eyre Basin in South Australia (interior desert regions of central and southern Australia) and across drought-affected regions in north-west NSW (Broken Hill and the red sand plains of western New South Wales), before arriving in the Sydney region. These long-range air parcels transported suspended dust particles, with high PM10 concentrations, over many areas of NSW. In this image, particulate matter at an altitude of 100 m (red line) entered the Sydney region from the north and northwest. The elevation of the particulate matter during the 72 h prior to the 22 November was below 100 m as it crossed the interior hot dry desert regions of central and south Australian where horizontal visibility was < 500 m. Particulate matter in this air mass arrived in the Sydney region still at an altitude of < 100 m.

The dust storm in November 2018 was similar to the red dust storm in 2009 that lifted about 2.54 million tons from surface soils and transported it towards, and beyond, the east coast where it caused economic damages totalling $A418–438 million (Tozer, 2012). Annually, two main trajectories transport dust over Australia. Bowler (1976) defined a northwesterly trajectory and a southeasterly trajectory and he suggested the centre of both dust storm routes pass across the Bathurst area. Sprigg (1982; Fig. 1) showed that various wind systems cause abrasion and transfer dust plumes along multiple paths across southern and eastern Australia. This dust is mainly deposited in the Pacific Ocean and Tasman Sea opposite the eastern coast of Australia (Shao et al., 2011).

Dust trajectories and sources during the Australian (Millennium Drought) decades (1990 to 2010) were analysed. The results displayed that dust events were more active during the spring and summer seasons in Australia. Usually, dust travel from central Australia towards the east coast of Australia in air cold mass cells from the Southern Ocean. O’Loingsigh et al. (2017) found three main wind trajectories transported the Australian dust from southerly Australia and northern NSW towards the east coast of Australia. These pathways include the northeastern trajectory of dust (25°S–20°S) caused by post-frontal winds blowing from the southwest; the eastern trajectory of dust (35°S–25°S) from Canberra and Sydney towards Brisbane caused by frontal winds blowing from the west; and the southern trajectory of dust (below 35°S) caused by prefrontal winds blowing from the north transferring dust towards the Victorian south coast.

## Conclusions

On 21 November 2018, atmospheric dust had reduced visibility to just metres in far western NSW including at Darling River and Broken Hill. By Thursday morning 22 November 2018, this orange dust storm had moved east stretched around 500-km wide and extended from Victoria to Queensland. It covered a large area of Sydney where PM10 concentrations were obtained from air quality monitoring stations before, during and after the occurrence of the dust storm over Sydney. The huge dust storm led to low visibility (< 500 m) in the eastern and western suburbs of Sydney. The PM10 values were below 50 μg/m^3^ prior to the dust storm but rose to 483 μg/m^3^ at Prospect in the northwest part of Sydney and a maximum PM10 of 327 μg/m^3^ at Randwick near the Sydney CBD. The PM10 values increased rapidly within a few hours after the arrival of the dust storm and then decreased to reach normal concentrations < 50 μg/m^3^ late in the day. During the day, these PM10 levels were considerably above the standard of air quality (50 μg/m^3^) in Australia. Variability of PM10 values in Sydney displayed the spatial variability of the dust storm. PM10 concentrations may also have urban and other particulate matter sources. When dust particles are below 10 μm in size, this usually means that they originated from remote dust sources.

In examined synoptic weather patterns, the location of low-pressure systems above southern and central areas of Australia causes the air masses to converge near the ground surface and diverge at higher levels. The negative vortex in southern Australia and the relatively strong positive vortex in western NSW leads to airflows and cyclonic motion from a northwestern direction. These higher wind velocities at low levels facilitate the transport of dust particles towards Sydney from the dry areas with inadequate vegetarian in western NSW. In general, the movement of troughs and cyclones over a wide region of southern NSW leads to dust pollution in the Sydney area. This is aided by the weakening of movement of high-pressure systems over the area in hot times. The dominance of thermal-low-pressure cells near the surface, especially in north-western NSW, is another major element leading to the occurrence of dust storms in the region. The results show that synoptic weather conditions (cold fronts, low pressure and high wind speed) cause the mobilisation and uplift of dust plumes into the atmosphere.

In the current study, sources and transport pathways of the 22 November dust storm over the Sydney region have been determined by backward trajectories using the HYSPLIT model. The dust storm began in western NSW and South Australia. Because of the high wind velocity associated with the cold front and low-pressure system, the dust plume was released from the desert areas and moved over much of NSW towards Sydney. The intensity of the dust storm on 22/11/2018 was due to the conjunction of the regional drought with high winds velocities. The drought conditions created wide semi-desert areas in the eastern part of Australia, especially in north-western NSW and the lower Lake Eyre Basin, where the dry surface soil and lack of vegetarian allowed extensive wind erosion. The transport pathway of dust from the HYSPLIT backward trajectories was confirmed by MODIS satellite images. In addition, the orange colour of the dust that entered the Sydney region meant that it had been transported a long distance (> 1100 km) from the semi-desert areas in Australia. The dust storm on 22 November was the largest and strongest dust event in spring 2018. The atmospheric conditions for this November 2018 dust storm were very similar to the atmospheric conditions that caused 23 September 2009 (red dawn) dust event, which also covered many parts of NSW and Sydney.

## Data Availability

Data available on request from the authors.
